# Biomonitoring of Mercury Contamination in Poland Based on Its Concentration in Scots Pine (*Pinus sylvestris* L.) Foliage

**DOI:** 10.3390/ijerph181910366

**Published:** 2021-10-01

**Authors:** Bartłomiej Woś, Piotr Gruba, Jarosław Socha, Marcin Pietrzykowski

**Affiliations:** 1 Department of Ecology and Silviculture, Faculty of Forestry, University of Agriculture in Krakow, al. 29 Listopada 46, 31-425 Krakow, Poland; piotr.gruba@urk.edu.pl (P.G.); marcin.pietrzykowski@ur.krakow.pl (M.P.); 2 Department of Forest Resources Management, Faculty of Forestry, University of Agriculture in Krakow, al. 29 Listopada 46, 31-425 Krakow, Poland; jaroslaw.socha@urk.edu.pl

**Keywords:** trace elements, mercury, pollution, biomonitoring, Scots pine

## Abstract

This work evaluates current mercury (Hg) contamination in Poland, represented by the Hg concentrations in Scots pine foliage. Samples were collected over 295 investigation plots in monitoring grids throughout Poland, from pines aged between 12 and 147 years. Analyses were conducted with consideration of bioclimatic factors and soil properties. Concentrations in the pine foliage did not exceed the values characteristic of an ecosystem unaffected by industrial pollution, ranging from 0.0032 to 0.0252 mg kg^−1^ dry mass. However, pine stands located in western and central Poland, and in the northwest near the Baltic Sea, exhibited higher Hg concentrations in foliage than in eastern regions. Hg content in foliage depends on the mean temperature of the driest quarter, as well as on Hg content in soils. This indicates that the periods of drought observed in recent years in Poland may affect Hg concentrations in pine foliage.

## 1. Introduction

Mercury (Hg) pollution is one of the most serious environmental problems, mainly due to its toxicity to environmental and human health [1]. Long-term Hg exposure can cause brain damage in humans, leading to metabolic disturbances, psychopathological symptoms, and sensory impairment [2].

Hg is emitted from natural sources—such as volcanic eruptions, ocean evaporation, geothermal processes, and weathering of minerals containing Hg [3,4]—or as a result of re-emission of Hg accumulated from preceding anthropogenic emissions [5,6]. However, the majority of Hg deposited in the environment comes from anthropogenic emissions [7]. In earlier years, it was estimated that as much as 70–80% of total Hg emissions into the environment were anthropogenic in origin [8]. As a result of international agreements, including the Minamata Convention on Mercury [5], the share of Hg deposition from anthropogenic sources is decreasing. Estimates in the year 2000 indicated that anthropogenic Hg emissions on a global scale are similar to Hg emissions from natural sources. Fossil fuel combustion—mainly coal—was the principal global contributor among anthropogenic sources, accounting for some 60% of Hg emissions to the atmosphere [9]. Currently, it is estimated that anthropogenic emissions account for ~30% of total global Hg emissions to the atmosphere, while 70% are from natural sources (primary Hg emissions and re-emissions) [7,10]. However, the global reservoir of atmospheric Hg has increased to 2–5 times above pre-industrial levels [11].

Poland’s Hg emissions are among the highest in Europe [12], due to the prominence of coal in the country’s energy mix [13]. The annual Hg emissions throughout Poland were estimated at 10 Mg in 2015 (about 0.03 kg per square kilometer), and they have remained nearly the same since 2000 [14]. In recent years, however, there has been a gradual decrease in Hg emissions which, in 2018, fell below 9 Mg. Poland’s share of overall European emissions in 2018 was 17.2% (a decrease of 1.2 percentage points compared to 2010) [15]. About half of Poland’s total Hg emissions were due to fossil fuel combustion for electricity, with the other main sources being processes in the cement, chemical, and metallurgical industries [9,10].

Due to the threats posed to ecosystems and human health, both local and global Hg monitoring are critical to meeting the international Hg treaty, as well as to most national policies [6]. One measure of Hg pollution and its spatial distribution is the biomonitoring and determination of Hg concentrations in plant tissues [16]. Biomonitoring can also inform environmental risk assessments by measuring or estimating the concentration of a pollutant in the environment, and then estimating stress on humans and the environment due to pollution exposure [17].

The Hg in plant foliage comes mainly from the air, because Hg uptake from the soil tends to be accumulated in the belowground biomass (i.e., the roots), and the translocation of this element above ground is poor [18,19]. For these reasons, the Hg concentration in the aboveground biomass of vascular plants is commonly used to assess air Hg pollution [17,20]. Climatic conditions such as temperature, light, wind, turbulence, and humidity affect the deposition and uptake of Hg by plants from the atmosphere [17]. However, one further mechanism of interest is foliar uptake of Hg^0^ volatilized from the soil. Soils have different potentials for Hg storage, depending on texture, soil organic matter content, and pH [21,22], so different soil potentials for Hg storage may affect Hg re-emission and volatilized values.

Scots pine (*Pinus sylvestris* L.) is one of the most commonly used tree species in biomonitoring in temperate climate zones, due to its wide geographical range, broad ecological amplitude, and high tolerance to pollution. A good indicator of environmental pollution is the pollutant content in foliage [23,24,25]. However, the use of pine needles to monitor mercury pollution has mainly been conducted on a regional scale—for example, in highly polluted regions [25,26]. The aim of this work is to determine Hg concentrations in Scots pine foliage in Poland in order to support the current biomonitoring of air pollution in Central and Eastern Europe. The following research hypotheses were tested: (1) Hg concentration in pine foliage overlaps with the spatial distribution of Hg emission and deposition in Poland, and is elevated in heavily urbanized regions; (2) Hg concentration in foliage depends on bioclimatic factors; and (3) Hg concentration in foliage depends on soil properties and parent rock material.

## 2. Study Sites

### 2.1. Location

**Figure 1:**
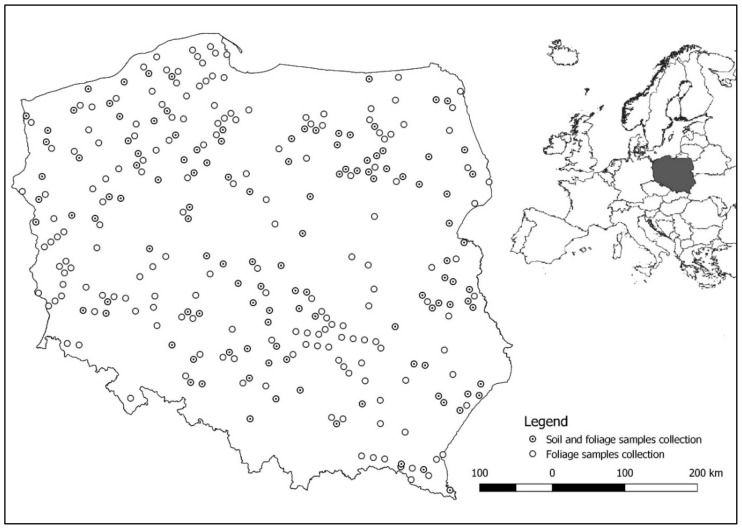
Location of monitoring plots for sampling of soil and Scots pine foliage.

The research was carried out at 295 permanent monitoring plots established throughout Poland (Figure 1). The plots were established in pure pine stands aged between 12 and 147 years (mean: 67 years). The area of the plot was selected so that each plot had a minimum of 30 trees, and ranged from 0.02 ha in the youngest (11–20 years old) to 0.10 ha in the oldest stands (> 80 years old) [27,28]. The distribution of research plots resulted from the diversity of habitat conditions, which is associated with the tree species compositions of stands. Scots pine in its natural range in Poland is mainly associated with lowland habitats [29].

### 2.2. Climate Characteristics

To characterize climate conditions, we estimated 19 bioclimatic variables for each plot using the WorldClim database [30]. The annual mean temperature (BIO1) for the research plots was ~7.6 °C (range from 5.5 to 9.1 °C). Temperatures ranged from a maximum of 23.1 °C in the warmest month (BIO5) to a minimum of 6.9 °C in the coldest (BIO6). Annual precipitation (BIO12) was 594 mm (from 499 to 779 mm). Precipitation in the wettest month (BIO13) was 80 mm and, for the driest month, 28 mm (BIO14; Table 1).

Growth and climate conditions were also described using topography. As an indirect measure of regional climate variations, we used elevation above sea level, estimated using a digital elevation model (DEM) with 25 m resolution and vertical accuracy (random mean square error) of +/− 7 m. Local microclimate was characterized by aspect and slope position. The local topographic wetness index (TWI) and slope position were derived from the DEM data [31]. Elevation above sea level was from 14.1 to 653.6 m. The slopes ranged from 0.0° to 14.2°. The research plots were characterized by a TWI between 5.40 and 16.19 (Table 2).

### 2.3. Soil Characteristics

The soils of the research plots were characterized by a diversity of parent rock types (GT: glacial tills; CS: claystones; MS: mudstones; L: loess; S: sands; SS: sandstones; LS: limestones; G: gravel) and groups (P: podzols; C: cambisols; M: mollisols; Le: leptosols; F: fluvisols; Lu: luvisols; BA: Brunic arenosols; CL: calcic leptosols) [28].

According to USDA soil classifications, heavy clay and sand were the most frequent soil types in the studied area. The pH of H_2_O in mineral layers (0–10, 10–40, and 40–100 cm) ranged from 3.5 to 8.7. Cation-exchange capacity (CEC) also showed a large variation, ranging from 0.76 to 60.77 cmol(+) kg^−1^. The carbon content in surface soil layers (0–10 cm) ranged from 0.3 to 47.7% [27,28,32]. The Hg content in soils was low, and within the typical range for unpolluted forest sites. The average Hg concentration in organic soil layers (O) was 0.12 mg kg^−1^; in 0–10 cm layers was 0.02 mg kg^−1^; and in both the 10–40 cm and 40–100 cm layers was 0.01 mg kg^−1^ (Table 3) [28].

## 3. Methods

### 3.1. Sampling and Laboratory Analyses

**Figure 2:**
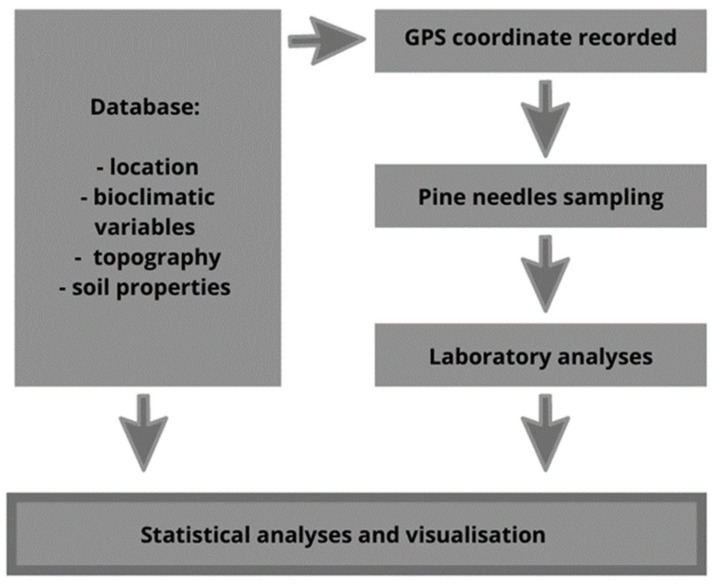
General scheme of the critical path for the data evaluation and study method.

On each site, one predominant tree (Kraft’s first class) was selected. From the first branch located in the southwest exposure of the crown, 25 pairs of one-year-old needles were collected from the seventh whorl, counting from the apex. The samples were taken during two vegetation seasons in 2015 and 2016 (Figure 2).

In the laboratory, the needle samples were dried at 65 °C, ground, and then the Hg content was determined using a DMA-80 Hg analyzer (Milestone), via drying and thermal decomposition [28]. Quality control was assured by calibration with standard European Reference Material (ERM) No CD281 (rye grass), with a certified Hg concentration of 0.0164 mg kg^−1^, and uncertainty of 0.0022 mg kg^−1^. The ERM analysis was performed at the beginning, and again at the end, of each experimental run (number of samples = 50). The ERM and each sample were measured twice, with acceptable differences between the measurements < 10% (recoveries above 90%).

### 3.2. Statistical Analyses and Visualization

The correlations between Hg concentration in pine foliage and bioclimatic variables, tree ages, and soil properties were analyzed using Pearson’s correlation matrix and the multiple forward stepwise regression method. The correlation coefficient, with n = 295 samples, was calculated at probabilities *p* = 0.05 and *p* = 0.01. The significance of individual independent variables in multiple regression equations was tested using the *t*-test at a significance level of *p* < 0.05. The statistical analyses were carried out using STATISTICA 13.1 software (StatSoft, Tulsa, OK, USA) (Figure 2).

Distribution of the investigated features was compared to the normal distribution using the Shapiro–Wilk test. The average values of analyzed characteristics of substrates were compared using ANOVA, preceded by Leven’s variance homogeneity test. Significant differences between mean values of Hg concentrations in pine foliage from different areas in Poland were tested by post hoc RIR Tukey’s multiple comparison procedure (at *p* = 0.05). The spatial interpolation analysis for visualization of Hg concentration distribution in pine foliage in Poland was performed using the IDW module of QGIS 3.10 software (QGIS Development Team) (Figure 2).

## 4. Results 

### 4.1. Spatial Variation in Hg Concentration

**Figure 3:**
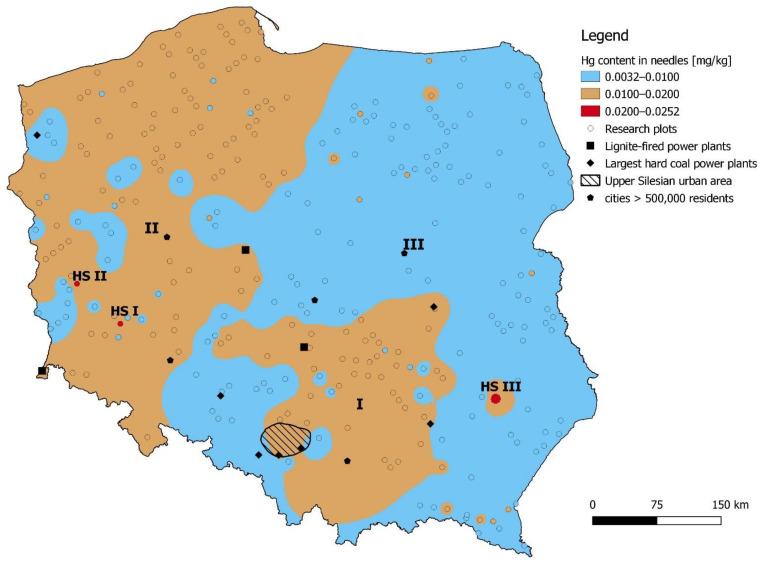
Spatial variation in Hg concentrations in pine foliage in Poland. Roman numerals indicate areas with different concentrations of Hg—I: central Poland; II: western Poland; III: remaining area with lower Hg concentrations up to 0.01 mg kg^−1^; HS I: hot spot I near Zielona Góra; HS II: hot spot II in the copper-exploitation region; HS III: hot spot III near Janów Lubelski.

Hg concentrations in Scots pine foliage ranged from 0.0032 mg kg^−1^ to 0.0252 mg kg^−1^ (mean: 0.01 mg kg^−1^). Relatively higher Hg concentrations (> 0.01 mg kg^−1^) were found in central (area I) and western Poland (area II). In addition, Hg concentrations in pine foliage exceeding 0.02 mg kg^−1^ were found in the vicinity of Zielona Góra (HS I), in the copper-exploitation region (HS II), and in the vicinity of Janów Lubelski (HS III) (Figure 3).

We determined that the average Hg concentrations in areas I (0.0128 mg kg^−1^) and II (0.0129 mg kg^−1^) were significantly higher than in area III (0.0075 mg kg^−1^) (Table 4).

### 4.2. Correlation between Hg Content in Pine Foliage, Bioclimatic Characteristics, and Soil Properties

Hg content in pine foliage correlated positively with the mean temperature of the driest quarter (BIO9), annual precipitation (BIO12), precipitation in the driest month (BIO14), precipitation in the driest quarter (BIO17), and precipitation in the coldest quarter (BIO19). Foliar Hg content correlated negatively with mean diurnal range (BIO2), temperature seasonality (BIO4), maximum temperature of the warmest month (BIO5), annual temperature range (BIO7), mean temperature of the wettest quarter (BIO8), and precipitation seasonality (BIO15; Table 5).

No significant correlation was found between foliar Hg content and stand ages or soil properties (Hg content, pH, texture, Corg, and CEC values). The lack of correlation with soil properties indicates that Hg content in pine needles does not depend on the soil parent material or geological strata, because different parent materials and geological strata have different properties. No significant correlation was found between foliar Hg content and elevation, aspect, slope position, or TWI index.

However, multiple regression analysis showed that foliar Hg content was related to the BIO9 and Hg content in the 0–100 cm soil layer. The determination coefficient explains 53% of the variation in foliar Hg content (Table 6; Equation (1)).

Hg in foliage (mg kg^−1^) = 0.001 × BIO9 (°C) + 0.157 × Hg_0–100_(mg kg^−1^) + 0.01 ± 0.002 (1)

## 5. Discussion

### 5.1. Hg Concentration and Spatial Distribution

Although Hg content in Scots pine foliage in Poland did not exceed the values found in ecosystems outside industrial emission areas, this study examined the spatial variation in mercury content across the country. According to Gworek et al. [33], plants growing beyond the influence of high Hg emissions had less than 0.1 mg kg^−1^ Hg. Higher Hg concentrations in pine foliage were found in areas affected by industrial pollution, including in the catchment area ~20 km from a lignite power plant in Spain (average 0.060 mg kg^−1^) [34], and the area around a chloralkali plant in the Czech Republic (from 0.0202 mg kg^−1^ in the plant’s low-impact zone, up to 0.0615 mg kg^−1^ in the high-impact zone) [35]. In urban areas of the Krakow agglomeration, pine foliage exhibited a higher concentration of Hg, ranging from 0.0286 to 0.0939 mg kg^−1^ [36]. However, Hg concentrations in pine foliage in areas affected by industrial pollution in Russia (in the basin of the Selenga River, within the boundaries of the Republic of Buryatia) ranged from 0.006 to 0.019 mg kg^−1^ [37], which fell within the range that we observed for Scots pine from Poland.

Spatial variation in Hg concentrations in Scots pine foliage is consistent with the estimates of spatial emission and deposition of Hg in Poland by Bartnicki et al. [14,38]. It can be assumed that a Hg concentration in pine foliage above 0.01 mg kg^−1^ overlaps with the emission levels estimated, for 2015, above 10 g km^−2^ year^−1^, and Hg deposition above 15 g km^−2^ year^−1^ [14]. However, on the basis of Hg concentration in Scots pine foliage, no “hot spots” were found in the Upper Silesia region, which has a significantly higher emission rate, exceeding 100 g km^−2^ year^−1^, and Hg deposition above 30 g km^−2^ year^−1^ [14,39]. There were also no higher Hg concentrations found in the vicinities of the largest cities (>500,000 inhabitants). The Hg concentrations in precipitation were often much higher at the urban sites compared to the research plots, which were all forest sites [40]. The absence of recorded hot spots in pine foliage may be due to the distribution of research plots, distance from emission sources, and scarcity of plots in this region. The spread of Hg from industrial sources varies depending on particle size and emission type. Larger particles fall nearby, while smaller particles are transported over long distances [17]. 

The highest Hg concentrations (>0.02 mg kg^−1^) in pine foliage occurred in three plots: The high Hg content in the first point (around Polkowice) resulted from the nearby Żelazny Most copper mining flotation waste landfill, 500 m to the south. The Żelazny Most flotation waste landfill is the largest of its kind in Europe, and has a negative impact on adjacent ecosystems through wind erosion and transport of pollutants—including trace elements—to both ground and surface waters [41]. In the case of the Legnica–Głogów Copper Mine District, it has already been reported by Barej et al. [42] that the Hg content in plant material (e.g., grasses and wheat cereals) exceeds those in unpolluted areas (0.1 mg kg^−1^ according to Gworek et al. [33]); however, the Hg concentrations found did not exceed the levels considered toxic, and the degree of contamination of plant material decreased over the 2002–2017 period [42,43]. The Hg concentrations in pine foliage in the vicinity of the flotation waste landfill in our study, although higher than those reported for the rest of Poland, also do not pose a threat to the environment. Similar values were given for the other two hot spots (around Zielona Góra and Janów Lubelski) located outside the areas with significant industrial emissions.

Interestingly, higher Hg concentrations in pine foliage were found in the vicinity of the Baltic Sea. Seas are an important sink for mercury deposition. Evaporation from the seas is one of the main factors of natural Hg emission to the environment, and also results from the re-emission of earlier depositions of anthropogenic origin. The sea is a Hg sink in winter, and an emission source during the growing season [44]. It was estimated in 2006 that total Hg emissions from the western and central parts of the Baltic Sea (235,000 km^2^, i.e., ~60% of the sea area) were 4300 ± 1600 kg, and accounted for less than 5% of Hg emissions across Europe. According to Kuss and Schneider [45], the highest Hg emissions from the Baltic Sea were found to occur in the summer, and the lowest were measured in winter, which is associated with evaporation from the sea during summer. In the southern part of the Baltic Sea in 1997 and 1998, the mean annual Hg flux was estimated to be 9.5 μg m^−2^ yr^−1^, and was higher in summer than in winter [46]. The decisive factor of Hg concentration in foliage should be considered the atmospheric Hg concentration. Although the Baltic Sea might act as an important Hg emission source, it does not exert a notable influence on atmospheric Hg concentrations [45]. The sea contributes humidity, and fog water (containing Hg) deposition would be an important source of Hg in foliage [47]. For these reasons, the sea may favor Hg uptake by plants through its summer evaporation, which coincides with the period of most intensive plant growth.

### 5.2. Impact of Bioclimatic Factors on Hg Concentration

Atmospheric Hg reaches plants via both dry and wet deposition [48,49]. Precipitation absorbs and removes aerosols and pollutants from the atmosphere, and pollutants from precipitation can be more easily taken up by plants than pollutants from dry deposition [50]. The correlation of Hg content in foliage with precipitation from the driest month (BIO14) and quarter (BIO17) may be due to the concentration of ions and pollutants in precipitation being highest during the driest quarter, rather than the wettest [50]. The multiple regression analysis showed that Hg content in foliage depends on the mean temperature of the driest quarter (BIO9) and Hg content in the 0–100 cm soil layer. This may be due to foliar uptake of Hg^0^ volatilized from the soil. The Hg content in the air often increased with increasing temperature, which was likely caused by temperature-dependent surface emissions [51]. The study of Smith-Downey et al. [52] estimated that 56% of Hg deposited in terrestrial ecosystems is re-emitted. High temperature also promotes the partition of the particle with reactive Hg to the gaseous phase [53]. However, Hg resources accumulated during previous industrial emissions in the environment are needed for this process [18]. At very high temperatures, energy production (and, thus, the emission of pollutants to the atmosphere) increases as a result of its demand for cooling devices [54]. This can also indicate that the periods of drought with high temperatures, observed in recent years in Poland, may affect Hg re-emissions from ecosystem accumulations due to previous anthropogenic emissions.

### 5.3. Impact of Soil Properties on Hg Concentration

No significant correlations were noted between Hg concentration in pine foliage and soil properties, including Hg content in soils, confirming the limited translocation of this element from the roots to the aboveground biomass [18,19]. Such localization also indicates the usefulness of determining Hg concentrations in foliage for assessing air pollution [17]. Earlier studies on the spatial variation in Hg contamination in Polish soils showed that Hg concentration in soils is highest in southern Poland, which includes mainly mountainous and foothill areas [28,55]. The spatial variability in Hg content in Scots pine foliage, however, does not match these relationships. Plants take up Hg directly from water or soil through root systems, or from the air through stomata [3,18]. However, Hg taken up by plants from the soil tends to accumulate in the roots, and tends to be bound in sulfhydryl (-SH) groups [18].

## 6. Conclusions

Hg concentrations in Scots pine foliage did not exceed the values typical for ecosystems without industrial pollution, which are considered non-threatening. Spatial variation in Hg concentrations in Scots pine foliage did not overlap with spatial variation in Hg contamination in Polish soils, but was consistent with the estimates of Hg emission and deposition. It can be assumed that Hg concentrations in pine foliage above 0.01 mg kg^−1^ overlap with the emission levels estimated for 2015 above 10 g km^−2^ year^−1^ and Hg deposition above 15 g km^−2^ year^−1^. However, based on Hg concentrations in Scots pine foliage, no “hot spots” were found in Upper Silesia, with its significantly higher emissions and the negative environmental effect of industrial pressure in recent years. Hg concentrations in pine foliage were positively correlated with the mean temperature of the driest quarter and annual precipitation. This correlation may indicate that Poland’s recent droughts may affect Hg re-emissions from stock accumulated in ecosystems from previous anthropogenic emissions.

## Figures and Tables

**Table 1:** Bioclimatic variables on research plots (*n* = 195; source of data: global climate and weather data, www.worldclim.org).

	Bioclimatic Variables ^1^																		
	BIO1 (°C)	BIO2 (°C)	BIO3 (%)	BIO4 (°C)	BIO5 (°C)	BIO6 (°C)	BIO7 (°C)	BIO8 (°C)	BIO9 (°C)	BIO10 (°C)	BIO11 (°C)	BIO12 (mm)	BIO13 (mm)	BIO14 (mm)	BIO15 (%)	BIO16 (mm)	BIO17 (mm)	BIO18 (mm)	BIO19 (mm)
Mean	7.6	8.0	26.2	7831	23.1	−6.9	30.1	17.1	−1.1	17.2	−3.0	594	80	28	34.0	223.0	91.8	222.6	103.4
Min.	5.5	6.2	23.0	6703	19.7	−10.0	24.8	14.7	−4.2	14.7	−5.3	507	64	20	22	178	71	178	75
Max.	9.1	9.1	31.0	8886	24.9	−3.9	33.2	18.5	3.1	18.5	−0.6	816	115	42	48	323	134	323	146
Max/Min	1.7	1.5	1.3	1.3	1.3	0.4	1.3	1.3	−0.7	1.3	0.1	1.6	1.8	2.1	2.2	1.8	1.9	1.8	1.9

^1^ BIO1: annual mean temperature; BIO2: mean diurnal range (mean of monthly (max temp−min temp)); BIO3: isothermality (BIO2/BIO7) (* 100); BIO4: temperature seasonality (standard deviation * 100); BIO5: maximum temperature of warmest month; BIO6: minimum temperature of coldest month; BIO7: annual temperature range (BIO5–BIO6); BIO8: mean temperature of wettest quarter; BIO9: mean temperature of driest quarter; BIO10: mean temperature of warmest quarter; BIO11: mean temperature of coldest quarter; BIO12: annual precipitation; BIO13: precipitation of wettest month; BIO14: precipitation of driest month; BIO15: precipitation seasonality (coefficient of variation); BIO16: precipitation of wettest quarter; BIO17: precipitation of driest quarter; BIO18: precipitation of warmest quarter; BIO19: precipitation of coldest quarter.

**Table 2:** Elevation, slope, aspect, and local topographic wetness index (TWI) of the research plots.

	Elevation (m)	Slope (°)	Aspect	TWI
Mean	167.6	2.4	199.21	8.33
Min.	14.1	0.0	2.01	5.40
Max.	653.6	14.2	359.82	16.19
Max/Min	46.5	-	179.22	3.00

**Table 3:** Selected soil properties on research plots [27,28,32].

Layer		pH in H_2_O	CEC	Corg	Particle Size Distribution			Hg
					Sand (2.0–0.05 mm)	Silt (0.05–0.002 mm)	Clay (< 0.002 mm)	
(cm)			(cmol(+) kg^−1^)	(%)				(mg kg^−1^)
O (litter)	Min.	3.3	-	46.4	-	-	-	0.00
	Max.	6.6	-	52.8	-	-	-	0.32
0–10	Min.	3.5	0.76	0.3	0	1	0	0.00
	Max.	7.0	59.49	47.7	98	81	36	0.36
10–40	Min.	3.6	0.11	0.1	0	1	0	0.00
	Max.	8.0	32.95	31.4	98	81	50	0.05
40–100	Min.	4.3	0.00	0.0	0	0	0	0.00
	Max.	8.7	60.77	4.1	100	87	53	0.08

**Table 4:** Hg content in pine foliage in designated areas, on the basis of interpolation.

Area	Hg (mg kg^−1^)			
	Mean	Min.	Max.	Max./Min.
I	0.0128 ± 0.0020 ^b 1^	0.0104	0.0180	1.73
II	0.0129 ± 0.0024 ^b^	0.0102	0.0180	1.77
III	0.0075 ± 0.0026 ^a^	0.0032	0.0099	3.09
HS I	0.0200	-	-	-
HS II	0.0208	-	-	-
HS III	0.0252	-	-	-

^1^ Mean ± SD; within columns, means followed by different letters (^a^, ^b^) are significantly different.

**Table 5:** Pearson’s correlation coefficients (r) between Hg content in pine foliage and bioclimatic variables.

	BIO1	BIO2	BIO3	BIO4	BIO5	BIO6	BIO7	BIO8	BIO9	BIO10	BIO11	BIO12	BIO13	BIO14	BIO15	BIO16	BIO17	BIO18	BIO19
Hg (mg kg^−1^)	0.09	−**0.32** **	−0.06	−**0.22** *	−**0.27** **	0.18	−**0.29** **	−**0.20** *	**0.44** **	−0.15	0.14	**0.27** **	0.15	**0.26** **	−**0.19** *	0.11	**0.28** **	0.09	**0.35** **

For explanation of abbreviations for bioclimatic variables, see Table 1. *: significant at *p* < 0.05; **: significant at *p* < 0.01.

**Table 6:** Regression summary for Hg content in pine foliage (mg kg^−1^) as the dependent variable, and BIO9 (°C) and Hg content (mg kg^−1^) in the 0–100 cm soil layer as independent variables; R^2^ adj. = 0.53; F (2.64) = 38.609; *p* < 0.0000.

Predictors	b	Standard Error of b	t(64)	*p*-Value
Intercept	0.01035	0.00044	23.54115	0.00000
BIO9	0.00105	0.00014	7.729090	0.00000
Hg_0–100_	0.15675	0.04025	3.894294	0.00024
